# Increased LGR6 Expression Sustains Long-Term Wnt Activation and Acquisition of Senescence in Epithelial Progenitors in Chronic Lung Diseases

**DOI:** 10.3390/cells10123437

**Published:** 2021-12-07

**Authors:** Emanuela E. Cortesi, Bob Meeusen, Arno Vanstapel, Stijn E. Verleden, Bart M. Vanaudenaerde, Wim A. Wuyts, Wim Janssens, Veerle Janssens, Tania Roskams, Juan-José Ventura

**Affiliations:** 1 Translational Cell & Tissue Research, Department of Imaging & Pathology, KU Leuven, 3000 Leuven, Belgium; tania.roskams@uzleuven.be; 2 Laboratory of Protein Phosphorylation and Proteomics, Department of Cellular and Molecular Medicine, KU Leuven, 3000 Leuven, Belgium; bob.meeusen@kuleuven.be (B.M.); veerle.janssens@kuleuven.be (V.J.); 3 Laboratory of Respiratory Diseases and Thoracic Surgery (BREATHE), Department of Chronic Diseases and Metabolism, KU Leuven, 3000 Leuven, Belgium; arno.vanstapel@kuleuven.be (A.V.); stijn.verleden@uantwerpen.be (S.E.V.); bart.vanaudenaerde@kuleuven.be (B.M.V.); wim.wuyts@kuleuven.be (W.A.W.); wim.janssens@kuleuven.be (W.J.); 4 Department of Pathology, UZ Leuven, 3000 Leuven, Belgium; 5 Department of Thoracic and Vascular Surgery & Pneumology, University Hospital Antwerp, 2650 Edegem, Belgium; 6 Antwerp Surgical Training, Anatomy and Research Centre (ASTARC), University of Antwerp, 2610 Wilrijk, Belgium

**Keywords:** LGR6, COPD, IPF, senescence, lung, progenitor cells

## Abstract

Chronic lung diseases (CLDs) represent a set of disorders characterized by the progressive loss of proper lung function. Among severe CLDs, the incidence of chronic obstructive pulmonary disease (COPD) and idiopathic pulmonary fibrosis (IPF) has grown over the last decades, mainly in the elderly population. Several studies have highlighted an increased expression of senescence-related markers in the resident progenitor cells in COPD and IPF, possibly undermining epithelial integrity and contributing to the progression and the aggravation of both diseases. Recently, the chronic activation of the canonical Wnt/β-catenin pathway was shown to induce cellular senescence. Here, we investigated the localization and the expression of leucin-rich repeat-containing G-protein-coupled receptor 6 (LGR6), a protein that activates and potentiates the canonical Wnt signalling. Through immunohistochemical analyses, we identified a lesion-associated rise in LGR6 levels in abnormal lung epithelial progenitors in COPD and IPF when compared to histologically normal tissues. Moreover, in areas of aberrant regeneration, chronic damage and fibrosis, LGR6-expressing epithelial progenitors displayed a major increase in the expression of senescence-associated markers. Our study suggests the involvement of LGR6 in the chronic activation of the Wnt/β-catenin pathway, mediating the impairment and exhaustion of epithelial progenitors in COPD and IPF.

## 1. Introduction

Aging is one of the main risk factors for developing chronic life-threatening conditions [1]. In the lung, aging is associated with structural and functional changes that increase susceptibility to chronic lung diseases (CLDs) [2,3]. Among CLDs, the prevalence of chronic obstructive pulmonary disease (COPD) and idiopathic pulmonary fibrosis (IPF) has been found to increase dramatically with age [4,5], with COPD being the third leading cause of death worldwide in 2019 [6]. Growing evidence suggests that anomalies in signalling pathways involved in lung morphogenesis and postnatal development may impact the integrity of epithelial progenitors in COPD and IPF [7,8]. Proliferation of resident bronchiolar and alveolar progenitors is critical for tissue regeneration in the light of re-establishing tissue homeostasis and maintaining correct lung functionalities [9]. Notably, bronchiolar regeneration is ascribed to club (CC10^+^) and basal (p63^+^, CK5^+^) progenitors that are capable of self-renewing and differentiating towards specialized ciliated and goblet cells [9]. In some circumstances, basal progenitors can migrate and proliferate in the alveolar compartment, where alveolar-type I (ATI) and -type II (ATII) cells are the sole resident epithelial cells. Beyond producing surfactant proteins, as surfactant protein-C (SPC^+^), ATII cells are the main alveolar progenitors and can differentiate into ATI cells and regenerate the distal lung epithelium [9]. Among other pathways, components of canonical and non-canonical Wnt signalling are expressed in the bronchial and in the alveolar progenitors of the adult lung [10]. In recent decades, the role of canonical Wnt signalling as a critical regulator for balancing stem cell proliferation and differentiation and as an inhibitor of apoptosis has been established for several human tissues and related diseases [11]. More specifically, in COPD and IPF, several studies highlighted the presence of aberrant Wnt activity [10,12,13,14,15]. Despite the fact that recent studies have provided new insights about the activation of the canonical Wnt pathway in IPF [15,16,17], the involvement of Wnt/β-catenin signalling in COPD is less clear. While previous findings suggested a shift from canonical to non-canonical Wnt pathway activation in emphysema [18], recent investigations showed that the Wnt/β-catenin pathway may be upregulated in the airway epithelium of patients with COPD [12,19]. 

In chronic diseases, the persistent activation of resident stem and progenitor cells can lead to stem cell exhaustion and senescence, resulting in reduced reparative capacities of tissues and organs [20,21]. Cells harbouring a senescent phenotype show an irreversible cell cycle arrest, mediated by increased levels of cell cycle inhibitors p16^INK4A^ and p21^CIP1^ and high senescence-associated β-galactosidase (SA-β-gal) enzymatic activity [22,23]. While senescence may initially support tissue repair [24], the accumulation of exhausted stem and progenitor cells may eventually impact the surrounding microenvironment and undermine effective tissue regeneration [1,25]. Recently, chronic Wnt pathway activation has also been associated with acquisition of a senescent phenotype by lung progenitor cells [26].

Previous studies conducted in human lungs revealed the existence of a bronchoalveolar multipotent progenitor population expressing the surface marker leucin-rich repeat-containing G-protein coupled receptor 6 (LGR6) [27]. As other members of the LGR family, LGR6 acts as a promoter of the canonical Wnt signalling [28] and its dynamic expression has been reported in epithelial stem and progenitor cells in several tissues, such as the skin, the bone and the mammary glands [29,30,31,32,33]. While ablation of LGR6-positive cells has detrimental consequences for the regeneration of skin, nails and bone, leading to defective tissue repair [34,35], increased LGR6 expression has been associated with tumour proliferation and invasion [30,34]. In the lung, due to their intrinsic capacity of differentiating into mature bronchiolar and alveolar cell types, LGR6-positive epithelial progenitors may be involved in the maintenance of lung homeostasis [27,36]. Moreover, a stage-related enrichment in LGR6-expressing cells was observed in advanced stages of lung adenocarcinoma, suggesting a role for LGR6-positive cells in lung cancer progression [37]. Considering the central role of Wnt pathway regulation in COPD and IPF and the importance of LGR6-expressing cells in other lung diseases, we currently investigated the localization and the expression of LGR6 in COPD and IPF lung tissues and its potential association with senescence of progenitor cells, a detrimental mechanism for epithelial barrier integrity and function. Our work suggests that increased LGR6 levels may foster the chronic activation of canonical Wnt/β-catenin signalling, which eventually results in the impairment and exhaustion of epithelial progenitor cells in both types of CLDs.

## 2. Materials and Methods

### 2.1. Samples Collection

All lung explants from donor and end-stage COPD and IPF patients (*n* = 29; Supplementary Table S1) were collected with informed consent, following the approval of the KU Leuven/UZ Leuven ethical committee (S52174 and S55886). Patients were recruited between January 2019 and September 2020. In 2020, following measures implemented at UZ Leuven, all recruited patients underwent COVID-19 RT-PCR tests and were found to be negative for SARS-CoV-2. Diagnoses for COPD and IPF have been performed according to GOLD and ATS/ERS consensus guidelines, respectively [38,39]. Despite being histologically normal, donor tissues were not used for transplantation due to non-pulmonary related reasons (lobar or single-lung transplantation, logistical issues, embolism, etc.). After excision, the use of samples was maximized to perform flow cytometry analyses, immunofluorescence and immunohistochemical evaluations. Briefly, portions of the explanted lungs were dissociated in enzymatic solutions (cfr. ‘Tissue Dissociation’), freshly frozen in liquid nitrogen-cooled isopentane and stored at −80 °C or fixed with 4% phosphate-buffered formaldehyde for at least 24 h.

### 2.2. Tissue Dissociation

Fresh tissues were thoroughly washed in cold Dulbecco’s Phosphate-Buffered Saline (D-PBS; Life Technologies, Paisley, UK, cat. no. 14190250) with antibiotics and antimycotics (Antibiotics-Antimycotics 100X; Life Technologies, 15240-062) to reduce fungal and bacterial contamination in the subsequent steps. Lung specimens were minced into fine pieces and transferred to fresh Advanced DMEM-F12 (Life Technologies, 12634028) supplemented with 1–3 mg/mL of collagenase type I, 1 mg/mL of dispase II (Life Technologies, 17100017 and 17105-041) and 1X PenStrep (Life Technologies, 15140122). The suspensions were incubated at 37 °C for 45–60 min and centrifuged at 1200 rpm for 5 min. The digested pellets were then resuspended in Advanced DMEM-F12 supplemented with 10% foetal bovine serum (FBS; Life technologies, 10500-064) to cease the enzymatic reactions. Samples were filtered through 70 μm cell strainers (Greiner Bio One, Frickenhausen, Germany, 542070) and serial centrifugation steps were performed at 1200 rpm for 5 min. Cell pellets were washed in D-PBS and treated with red blood cell lysis buffer (Roche, Mannheim, Germany, 11814389001) for 10 min at room temperature. After centrifugation, isolated cells were washed in D-PBS and preserved for further analyses.

### 2.3. Immunohistochemical Stainings

Formalin-fixed and paraffin-embedded (FFPE) serial sections of 4 μm were cut at the microtome and transferred on Leica microscope slides (Leica Microsystems, Wetzlar, Germany, S21.2113.A). Slides were dried and haematoxylin and eosin stainings were performed. Sections for each specimen were selected for further immunohistochemical (IHC) and immunofluorescent (IF) investigations. Staining procedures were conducted using an automated IHC system (Bond™ Max, Leica Biosystems, Nussloch, Germany) according to the manufacturer’s instructions. Primary antibodies and respective dilutions used for the non-automated staining procedures are listed in Supplementary Table S2. Secondary anti-rabbit and anti-mouse HRP-conjugated antibodies were obtained from Dako (Agilent, Glostrup, Denmark, K400311-2 and K400111-2). 3,3′-diaminobenzidine solution (DAB; Liquid DAB^+^ Substrate Chromogen System, Dako, K346889-2) was used as a chromogen to detect antigen–antibody complexes. After staining, slides were washed and mounted using a Leica CV5030 Glass Coverslipper machine (Leica Biosystems) and pictures were acquired with a Leica DM2000 Histology Microscope (Leica Microsystems, RRID: SCR_020223). Stainings were quantified with QuPath (version 0.2.3, University of Edinburgh, Edinburgh, UK, RRID: SCR_018257), and non-parametric ANOVA with Dunn’s post hoc test was performed with Anaconda Software (version 1.9.7, Anaconda Inc., Austin, TX, USA). Values of *p* < 0.05 were considered significant.

### 2.4. Immunofluorescence Analyses

Tissue slides were deparaffinized in a Leica Autostainer XL Automated Slide Stainer (Leica Microsystems, RRID: SCR_020212) and heat-induced epitope retrieval was performed using a citrate buffer (EnVision FLEX Target Retrieval Solution Low pH, Dako, K8005) in a PT Link module (Dako), following manufacturer’s instructions. Lung sections were blocked with 5% bovine serum albumin (BSA, Roche, 03116956001) for at least 30 min at room temperature and incubated at 4 °C overnight with primary antibodies (Supplementary Table S2). After washing, Alexa Fluor^®^ 488 and 647 secondary antibodies (1:300, Jackson ImmunoResearch Europe Ltd., Ely, UK, 711-545-149, 715-545-150, 711-605-149 and 715-605-150) were applied onto the sections for 45–60 min. Slides were mounted using a ProLong™ Gold Antifade Mountant with DAPI (Thermo Fisher Scientific, Eugene, OR, USA, P36931) and IF analyses were carried out using a Leica DM2000 microscope (Leica Microsystems).

### 2.5. SA-β-Galactosidase Stainings

Senescence-associated β-Galactosidase (SA-β-Gal) is a hydrolytic enzyme residing in lysosomes, where it converts β-galactosides into monosaccharides. The enzymatic activity of SA-β-Gal is increased when cells become senescent, and its expression has been correlated with cellular aging and senescence in cultured cells and in tissues [40]. SA-β-Gal staining was performed on frozen sections using a Senescence β-Galactosidase Staining Kit (Cell Signaling Technology, Danvers, MA, USA, #9860). Lung tissues were preserved in OCT (KP Cryo-compound; VWR, Gavere, Belgium, K1620-C), frozen in liquid nitrogen-cooled isopentane (2-Methylbutane; Acros Organics, Geel, Belgium, AC126470010) and stored at −80 °C. Frozen sections were obtained using a Cryostar NX70 (Thermo Scientific, Waltham, MA, USA) and transferred on microscope slides. Samples were fixed in 1X fixative solution for 10 min at room temperature and rinsed twice with D-PBS. β-Galactosidase staining solution was freshly prepared according to the manufacturer’s instruction and applied on the sections. Lung sections were incubated at 37 °C overnight in a dry incubator in absence of CO_2_. On the following day, tissues were checked under a microscope for the development of blue colour and stored at 4 °C with a layer of 70% glycerol solution (Glycergel^®^, Dako, C0563), before image acquisition.

### 2.6. Flow Cytometry Analyses

Isolated human lung cells were stained for SA-β-Gal using the Cell Event™ Senescence Green Flow Cytometry Assay Kit (Invitrogen™, Thermo Fisher Scientific, C10840), following manufacturer’s instructions. Briefly, after dissociation, lung cells were fixed in 2% PFA (Thermo Scientific, 28908) for 10 min at room temperature. Cells were washed, centrifuged, and resuspended in freshly prepared working solution and incubated for 1–2 h at 37 °C in a dry incubator, protected from the light. After incubation, cell suspensions were washed twice with 1% BSA in D-PBS and permeabilized with 0.1% Tween20^®^ (Sigma, St. Quentin Fallavier, France, P1379) in D-PBS for 15 min on ice. After washing, cells were incubated with anti-LGR6 antibody (1:100; Abcam, Waltham, MA, USA, ab126747) and with APC-conjugated anti-human CD45 and CD31 antibodies (1:1000 and 1:1500; BioLegend, San Diego, CA, USA, 304012 and 303116) for 60 min on ice. Secondary DyLight™ 405 donkey anti-rabbit (1:500; Jackson ImmunoResearch Europe Ltd., 711-475-152) was used to detect LGR6 expression. Samples were run on BD Scientific Canto II Flow Cytometer (BD Bioscience, San Diego, CA, USA, RRID: SCR_018056) and flow cytometry data were analysed with FCS Express™ (version 7, DeNovo Software, Glendale, CA, USA, RRID: SCR_016431).

### 2.7. TUNEL Assay

TUNEL assay was performed on FFPE tissue slides using Click-iT™ Plus TUNEL Assay kit (Invitrogen™, C10617) according to the manufacturer’s instructions. After deparaffinization of tissue sections, slides were fixed in 4% PFA (Thermo Fisher, Kandel, Germany, 043368.9M) for 15 min at 37 °C and washed in PBS. Tissue sections were covered with permeabilization reagent, incubated for 15 min, and washed in D-PBS before being incubated with 4% PFA for further 5 min at 37 °C. After pre-treatment with the reaction buffer, TdT enzymatic solution was prepared following the provided instructions and allowed to incubate with tissue sections for 60 min at 37 °C. Slides were rinsed in deionized water, washed with 0.1% of Triton™ X-100 (Sigma, Burlington, MA, USA, T8787) + 3% BSA in PBS for 5 min. After rinsing with PBS, tissues were incubated with freshly prepared Click-iT™ TUNEL Reaction cocktail for 30 min at 37 °C, protected from the light. Slides were washed and mounted with ProLong™ Gold Antifade Mountant with DAPI and IF analyses were carried out using a Leica DM2000 fluorescent microscope.

## 3. Results

### 3.1. LGR6 Expression Is Increased in Fibrotic and Inflated Lesions and in Areas of Bronchiolization in COPD and IPF Samples

**Figure 1:**
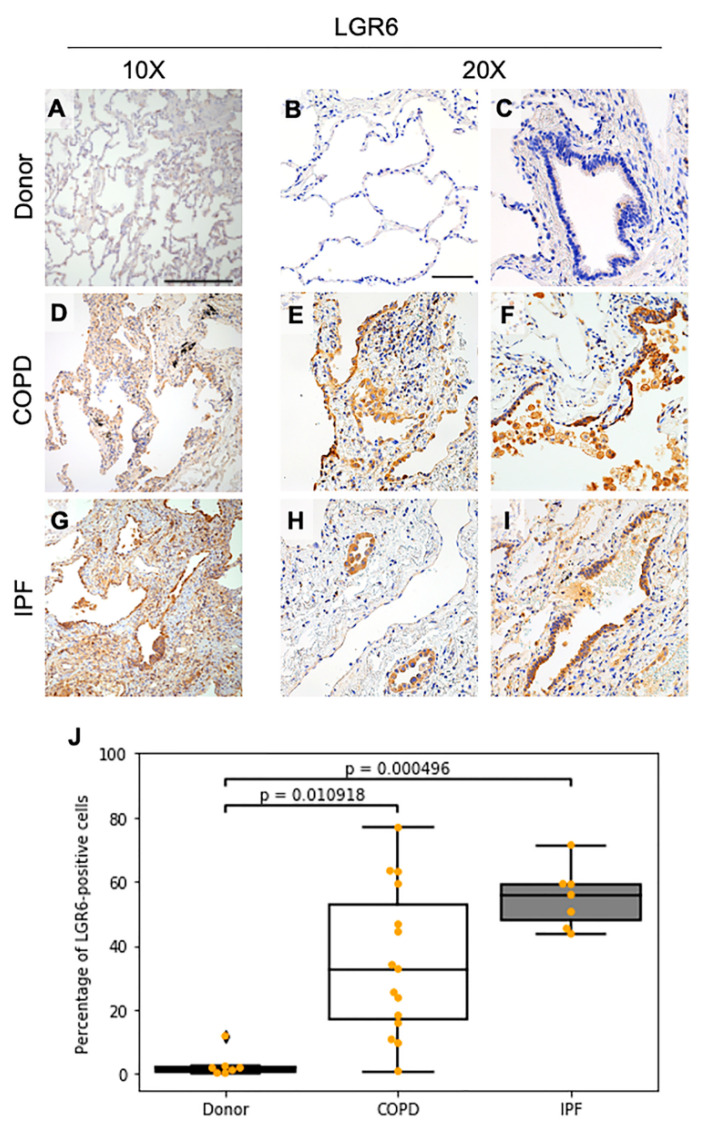
Immunohistochemical stainings depict high LGR6 protein levels in COPD and IPF tissues. (**A**–**I**): Immunohistochemical images representative of human donor, COPD and IPF lungs stained at 10× (**A**,**D**,**G**; scale bar = 300 μm) and at 20× (bar = 100 μm) magnification to detect localization of LGR6 in alveolar (**B**,**E**,**H**) and bronchiolar (**C**,**F**,**H**) areas. (**J**): Quantification of LGR6-positive cells for donor (*n* = 7), COPD (*n* = 15) and IPF (*n* = 7) tissues. Statistics: non-parametric ANOVA (*p*^donor vs. COPD^ = 0.010918, *p*^donor vs. IPF^ = 0.010918 and *p*^COPD vs. IPF^ = 0.408217).

We first investigated the intracellular expression of leucine-rich repeat-containing G-protein-coupled receptor 6 (LGR6) in donor (*n* = 7), COPD (*n* = 15) and IPF (*n* = 7) lungs (Figure 1 and Supplementary Table S1). In normal human lungs, no to low intracellular LGR6 expression was observed, and if present, it was limited to the apical portion of few bronchiolar cells and to perivascular and peribronchiolar pneumocytes, as assessed through morphological and immunophenotypical analyses on serial sections (Figure 1A–C).

We observed distinguishable patterns of expression for LGR6 in lungs obtained from patients with COPD. While emphysematous alveolar regions were completely negative (Supplementary Figure S1A), medium to high intracellular LGR6 expression was observed in fibrotic alveolar areas harbouring thick alveolar walls and inflammation (Figure 1D,E). To a similar extent, increased LGR6 levels were reported in narrowed airways and damaged bronchioles with evident loss of bronchiolar cells or expansion of bronchiolar cells towards the alveolar regions (Figure 1F).

In samples obtained from patients diagnosed with severe IPF, we observed a more homogeneously distributed, high intracellular LGR6 expression in all epithelial progenitors, with high protein concentration in disrupted airways as well as in alveolar areas (Figure 1G–I).

In diseased tissues, LGR6 staining was also detected to a variable extent in immune cells, reaching high levels in alveolar and interstitial macrophages in COPD and IPF samples (Supplementary Figure S1B,C). While the expression of LGR6 has been reported before in human phagocytes, albeit its role is not completely explored, and to our knowledge LGR6 expression has not yet been documented in other immune populations, we observed increased LGR6 levels in alveolar and interstitial macrophages and in lymphoid follicles (Supplementary Figure S1B–D) of COPD and IPF lungs.

Overall, lung specimens from patients with COPD and IPF showed a significant increment in the number of LGR6-positive (LGR6^+^) cells when compared to donor tissues (Figure 1J; *p*^donor vs. COPD^ = 0.010918 and *p*^donor vs. IPF^ = 0.000496).

### 3.2. LGR6 Is Highly Expressed in Basal, Club and Alveolar Type II Progenitors Localized in Damaged Bronchioles and Fibrotic Alveoli

**Figure 2:**
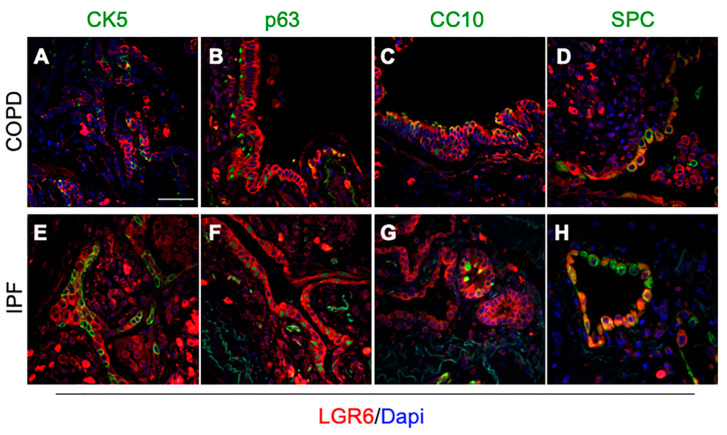
Increased LGR6 expression is observed in epithelial progenitor cells. (**A**–**H**): Immunofluorescence analyses depicted co-expression of LGR6 (red) and main epithelial progenitor markers (green). CK5 (**A**,**E**; bar = 50 μm) and p63 (**B**,**F**) markers were used to identify basal progenitors, CC10 stained club cells (**C**,**G**) and SPC was used to recognize ATII cells (**E**,**H**).

Previous studies conducted on epithelial progenitors in human lung tissues proved the existence of a LGR6^+^ progenitor population: LGR6^+^ epithelial progenitors are responsible for the homeostatic maintenance of the bronchoalveolar epithelium [27] and are involved in the lung adenocarcinoma progression [37]. Similarly, in donor tissues we observed sporadic and low intracellular LGR6 expression in few epithelial cells (Figure 1A). We further investigated the expression of LGR6 protein in epithelial progenitors of COPD and IPF (Figure 2A–H), where the bronchiolar and alveolar compartments showed prominent rearrangements, with epithelial progenitors harbouring manifest morphological alterations (Supplementary Figure S2A–H). To determine whether LGR6 positivity correlated with a specific population, we performed co-stainings between LGR6 and cell type specific markers for ATII cells (SPC^+^), club (CC10^+^) and basal (p63^+^ and CK5^+^) progenitors (Figure 2 and Supplementary Figure S3).

In COPD tissues, club (CC10^+^) and basal cells (p63^+^ and CK5^+^) expressing LGR6 were observed in narrowed airways and damaged bronchioles (Figure 2A–C). Moreover, intracellular LGR6 also showed a trend of increased expression at the interphase between bronchioles and alveoli, where club (CC10^+^) and basal progenitors (p63^+^ and CK5^+^) expanded towards the alveolar region (Supplementary Figure S3E–G). In the alveolar compartment, while ATII cells (SPC^+^) of emphysematous areas were negative for LGR6, we identified niches of morphologically abnormal LGR6^+^/SPC^+^ ATII cells in proximity to fibrotic lesions (Figure 2D and Supplementary Figure S3H).

A general expansion of the bronchiolar regions and a nearly complete loss of the alveolar compartment were observed in IPF tissues (Supplementary Figure S2E–H). Similar to proliferating bronchiolar progenitors in COPD samples, the expression of LGR6 was increased in basal (p63^+^ and CK5^+^), club (CC10^+^) and ATII (SPC^+^) cells (Figure 2E–H and Supplementary Figure S3I–L). Interestingly, at the bronchiole–alveoli interphase, we detected narrowed alveolar regions with co-existing LGR6^+^/SPC^+^ and LGR6^+^/p63^+^/CK5^-^ cells (Supplementary Figure S4D–F), with low to medium SPC levels and weak p63 nuclear positivity, as observed in immunohistochemical stainings (Supplementary Figure S4A–C). Further immunofluorescent analyses confirmed the consistent co-expression between basal and ATII markers in some cells of these regions (Supplementary Figure S4G–J). Despite the contiguity of the respiratory bronchioles with the alveolar compartment, co-expression of basal (p63^+^) and ATII (SPC^+^) markers were rarely observed in COPD samples (Supplementary Figure S4K–N).

### 3.3. In Fibrotic and Inflated Areas, Senescent Progenitor Cells Show Increased LGR6 Expression

**Figure 3:**
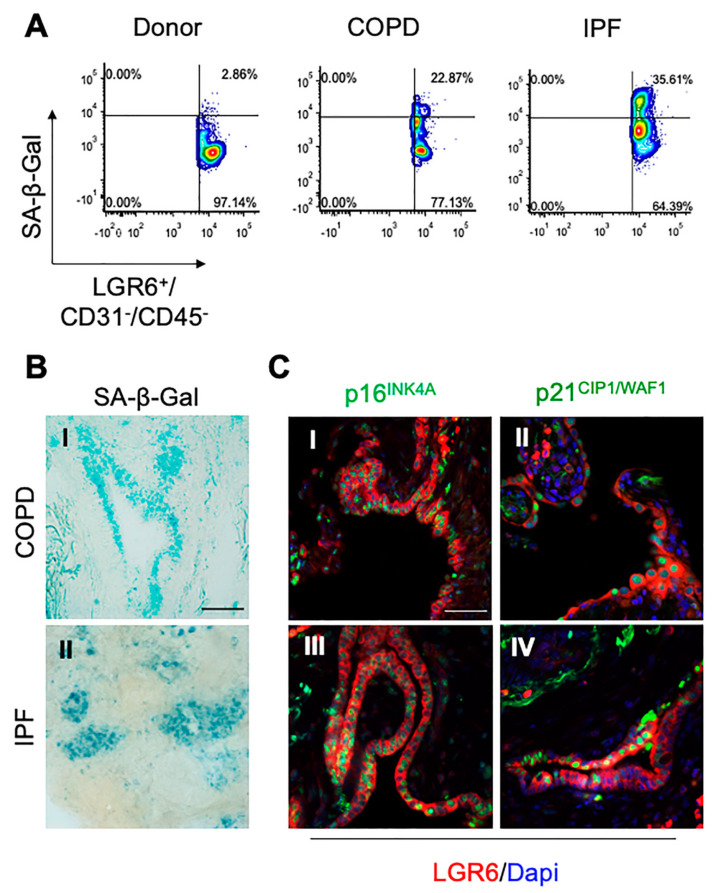
LGR6+ cells express increased levels of senescence-associated markers. (**A**,**B**) SA-β-Gal enzymatic activity was evaluated in donor, COPD and IPF cells isolated from freshly dissected lung biopsies (**A**). SA-β-Gal expression was confirmed in bronchoalveolar compartment of COPD and IPF tissues ((**B**); bar = 100 μm). (**C**) Representative photomicrographs showing co-expression of LGR6 in red with p16INK4A ((**I**)–(**III**)) and p21CIP1/WAF1 ((**II**)–(**IV**)) in green in the bronchoalveolar compartment of COPD and IPF patients ((**C**); scale bar = 50 μm).

Signs of physiological lung aging have been previously reported in COPD and IPF tissues [23]. Moreover, increased Wnt signalling has been associated with accelerated aging and induction of cellular senescence [26,41]. Considering the role of LGR6 as an enhancer of the canonical Wnt pathway, we investigated the expression of senescence-associated markers in LGR6^+^ epithelial progenitors. While we did not observe the nuclear expression of proliferation marker Ki67 (Supplementary Figure S5A–F) and we excluded the presence of apoptotic events occurring in COPD and IPF tissues (Supplementary Figure S5K–N), we detected increased levels of Wnt-related markers total β-catenin and glutamine synthetase in lesions harbouring LGR6+ progenitors (Supplementary Figure S5G–J). Using flow cytometric analyses, we evaluated changes in the enzymatic activity of senescence-associated β-Galactosidase (SA-β-Gal) in cells obtained from control, COPD and IPF biopsies. After negative selection for CD45 and CD31 markers to avoid hematopoietic and endothelial contamination, we assessed the expression for SA-β-Gal in LGR6+ cells. As shown in Figure 3A, we observed an increased SA-β-Gal enzymatic activity in LGR6+ cells obtained from dissociated COPD and IPF specimens, when compared to cells isolated from healthy donor biopsies.

We further confirmed the expression of SA-β-Gal in COPD and IPF frozen sections (Figure 3B). Immunohistochemical analyses showed increased levels of expression of cell cycle markers p16INK4A and p21CIP1 (Figure 3C and Supplementary Figure S6), which are routinely used as markers for senescent cells [42].

In COPD samples, some niches of morphologically abnormal LGR6+ epithelial progenitors with altered morphology showed increased p16INK4A and/or p21CIP1 expression (Figure 3CI,II and Supplementary Figure S6C,D). LGR6+ progenitors expressing senescence-associated markers were found in proximity to fibrotic alveolar areas and damaged bronchioles. Further investigations revealed that LGR6+ cells of damaged areas were also SPC+ and CC10+ cells expressing p16INK4A and/or p21CIP1 (Supplementary Figure S7E–H). While sporadic senescent LGR6+/p63+ cells were observed in few COPD samples, senescence-associated markers were not expressed in CK5+ or p63+ basal progenitors of thick peripheral airways (Supplementary Figure S7A–D).

The expression of senescence-related markers was increased in LGR6+ epithelial progenitors throughout all IPF tissues (Figure 3CIII,IV and Supplementary Figure S6E,F). High levels of p16INK4A and p21CIP1 were reported in LGR6+/SPC+ and LGR6+/CC10+ progenitors. The expression of p16INK4A and p21CIP1 was more diffuse in the LGR6+/CK5+ basal population than in LGR6+/p63+ cells.

Our studies show that, in end-stage COPD and IPF tissues, bronchoalveolar epithelial progenitor cells display a lesion-associated rise of LGR6 levels, which is followed by an overall increased expression of canonical Wnt-related markers, total β-catenin and glutamine synthetase and by the expression of senescence-associated markers p21 and p16 in areas of aberrant tissue regeneration.

## 4. Discussion

Although current treatments can relieve symptoms and delay the progression of the two diseases, COPD and IPF remain progressive and incurable conditions [38,39]. Our understanding of pivotal mechanisms involved in CLDs has improved in recent decades, however, not to the complete elucidation of the complex machinery contributing to COPD and IPF development and exacerbation [7,8,43,44,45,46,47].

Among all proposed pathways, increasing interest has been raised around the activity of Wnt signalling in CLDs. Whilst in patients with IPF, the involvement of Wnt/β-catenin signalling has been widely investigated in recent decades [15,16,17], in COPD the activation of canonical Wnt pathway is still debated and its upregulation may be lesion specific [12,18,19]. In our study, we explored the expression and localization of LGR6, a known enhancer of the canonical Wnt signalling that has been previously shown to contribute to lung adenocarcinoma progression [37]. In lungs with COPD and IPF, we found increased intracellular LGR6 expression in epithelial niches of fibrotic lesions and proximal to parenchymal and peribronchiolar lymphoid follicles. In contrast, LGR6 was absent in normal lungs and in emphysematous regions of COPD tissues. Whilst bronchiolar and alveolar fibrotic lesions showed a corresponding increase in LGR6 levels in club and ATII cells, the pattern of LGR6 expression in basal progenitors of IPF and COPD was more heterogeneous. In IPF samples, LGR6 expression was generally increased in the basal progenitor populations, reaching the highest protein levels in areas of bronchiolization and honeycombing, whereas in COPD tissues, LGR6-positive basal cells were observed in damaged and inflated bronchioles and in proliferative bronchiolar lesions, but not in airways proximal to emphysematous lesions. The presence of LGR6 in lung epithelial progenitors of damaged and proliferating bronchioles and in fibrotic alveoli may suggest a Wnt-mediated response in divergent chronic insults occurring in COPD and IPF [10,46,48].

Aberrant Wnt/β-catenin signalling has been widely reported in aging-related diseases and in fibrosis in several tissues, including human lungs [15,25,43,49]. Recent studies showed that chronic activation of canonical Wnt signalling may contribute to progenitor exhaustion through induction of senescence [26,49,50]. Senescence is considered one of the main hallmarks of aging and accumulation of senescent cells and secretion of senescence-related factors (SASPs) may contribute to exacerbation, inflammation and failure of tissue regeneration [9,23]. Alongside the increased expression of LGR6 in epithelial progenitors of COPD and IPF tissues, we observed higher senescence-associated β-galactosidase (SA-β-Gal) activity and increased p16^INK4A^ and p21^CIP1^ levels in LGR6-expressing cells. Interestingly, while p16^INK4A^ expression was homogenously increased in all epithelial progenitors, p21^CIP1^ staining was limited to some regions harbouring evident fibrotic lesions, tissue damage and/or defective bronchiolization. As recently published by Lehmann et al. [26], while an increase in p16^INK4A^ levels tends to occur early and may not be sufficient to induce a manifest senescent phenotype, accumulation of p21^CIP1^ occurs later in time, in parallel with rising SA-β-Gal activity. According to these observations, our findings may suggest that LGR6-positive ATII and a few club and basal progenitors harbouring high p21^CIP1^ levels may display an actual senescent phenotype in IPF. In contrast, in COPD the presence of senescent LGR6-positive ATII cells may be limited to some niches of the alveolar compartment, where evident parenchymal fibrosis and senescence may be extended to sporadic LGR6-expressing club and basal progenitors in areas of defective bronchiolization.

While activation of the canonical Wnt pathway has been proposed as a supporting mechanism for tissue regeneration [11], aberrant Wnt activation may lead to induction of a senescent phenotype [26,49]. R-spondins (RSPOs) can elicit the activation of the Wnt pathway through binding with LGR receptors [11]. Recently, some studies have reported the accumulation of RSPO2 and RSPO3 in fibroblasts and epithelial cells of fibrotic lungs, supporting their potential as molecular targets [51,52] and, in light of our work, a newfound place for LGR6 in mediating fibrotic diseases. In parallel, the impairment of the phagocytic and the efferocytic activities for human macrophages has been reported in COPD and IPF, respectively [53,54]. Recent findings highlighted the capacity of LGR6 in stimulating innate immune response in human phagocytes [55] through maresin1 interaction, suggesting a new potential function for LGR6 in resolving inflammation and supporting wound healing. All these breakthroughs may imply a novel role for LGR6 in physiological and pathological conditions that will require further investigation.

Our data indicate the involvement of LGR6 in CLDs, suggesting a pro-regenerative LGR6-mediated activation of canonical Wnt/β-catenin pathway in COPD and IPF, which ultimately results in a chronic signalling that fosters the acquisition of a senescent phenotype and the exhaustion of lung epithelial progenitors. Further in vitro studies would be needed to elucidate the precise underlying mechanisms.

## Data Availability

All data analyzed during this study are included in this published article and its Supplemetary Material section.

## Supplementary Materials

The following are available online at https://www.mdpi.com/article/10.3390/cells10123437/s1, Table S1: Demographic data and smoking history of all recruited patients; Supplementary Table S2: List of antibodies; Supplementary Figure S1: Expression of LGR6 in the tissue environment; Supplementary Figure S2: Alteration of progenitor cells niches in the lungs with COPD and IPF; Supplementary Figure S3: LGR6 expression in progenitor populations; Supplementary figure S4: LGR6 in alveolar and basal progenitors; Supplementary Figure S5: Non proliferating LGR6+ cells express markers of senescence and Wnt pathway activation, but not markers of apoptosis; Supplementary Figure S6: Senescent LGR6+ cells accumulate in COPD and IPF tissues; Supplementary Figure S7: Lung epithelial progenitors show expression of senescence-associated markers.
