# Supplementary material for: Increased LGR6 Expression Sustains Long-Term Wnt Activation and Acquisition of Senescence in Epithelial Progenitors in Chronic Lung Diseases
Source: Cells. 2021 Dec 7;10(12):3437. doi: 10.3390/cells10123437 (PMC8700573; doi:10.3390/cells10123437)
Supplement: Supplementary file 1 [file cells-10-03437-s001.zip › cells-1456286-supplementary.pdf]

cells MDPI

Supplementary Material to Cortesi et al., "Increased LGR6 expression sustains long-term Wnt activation and acquisition of senescence in epithelial progenitors in chronic lung diseases".

All figures and tables are cited in the main text.

Table S1. Demographic data and smoking history of all recruited patients. Data are reported as mean ± SD. Patients with COPD and IPF were all end-stage cases. *F*, female; *M*, male; *ns*, non-smoker; *es*, ever smoker; *u*, unknown; COPD, Chronic Obstructive Pulmonary Disease; IPF, Idiopathic Pulmonary Fibrosis.

		Donors	COPD	IPF
**N.**		7	15	7
**Sex**	*F*	1	6	3
	M	6	9	4
**Age**		45,43 ± 16,97	59,4 ± 5,55	63,86 ± 1,68
**Smocking status**	*ns*	4	0	0
	es	1	15	7
	u	2	0	0
**Pack year**		*es*= 10	31,87 ± 16,7	23,29 ± 12,8

Table S2. List of antibodies. Primary antibodies used for manual immunohistochemical and immunofluorescent stainings are listed in alphabetical order.

Protein	Clone	Host and isotype	Ref.	Brand	Dilui-tion
**CC10**	E-11	mouse IgG1	sc-365992	Santa Cruz, Heidelberg, Germany	1:500
	H-75	rabbit IgG	sc-25554	Santa Cruz, Heidelberg, Germany	1:500
**CK5**	XM26	mouse IgG1	NCL-L-CK5	Novocastra, Newcastle upon Tyne, UK	1:100
**Glutamine synthetase**	6/Glutamine Synthetase	mouse IgG2a	610518	BD Biosciences, San Diego, CA, USA	1:400
**KI67**	MIB-1	mouse IgG1	IR626	Dako, Glostrup, Denmark	ready-to-use
**LGR6**	EPR6874	rabbit IgG	ab126747	Abcam, Waltham, MA, USA	1:150
**p16^{INK4A}**	2D9A12	mouse IgG2b	ab54210	Abcam, Waltham, MA, USA	1:2000
**p21$^{CIP1/WAF1}$**	SX118	mouse IgG1	M720229-2	Dako, Glostrup, Denmark	1:30
	C-19	rabbit IgG	sc-397	Santa Cruz, Heidelberg, Germany	1:20
**p63**	DAK-p63	mouse IgG2a	M7317	Dako, Glostrup, Denmark	1:30
**SPC**	H-8	mouse IgG2b	sc-518029	Santa Cruz, Heidelberg, Germany	1:500

	---	rabbit IgG	AB3786	Merck, Darmstadt, Germany	1:500
**Total β-catenin**	14/Beta-Catenin	mouse IgG1	610154	BD Biosciences, San Diego, CA, USA	1:200

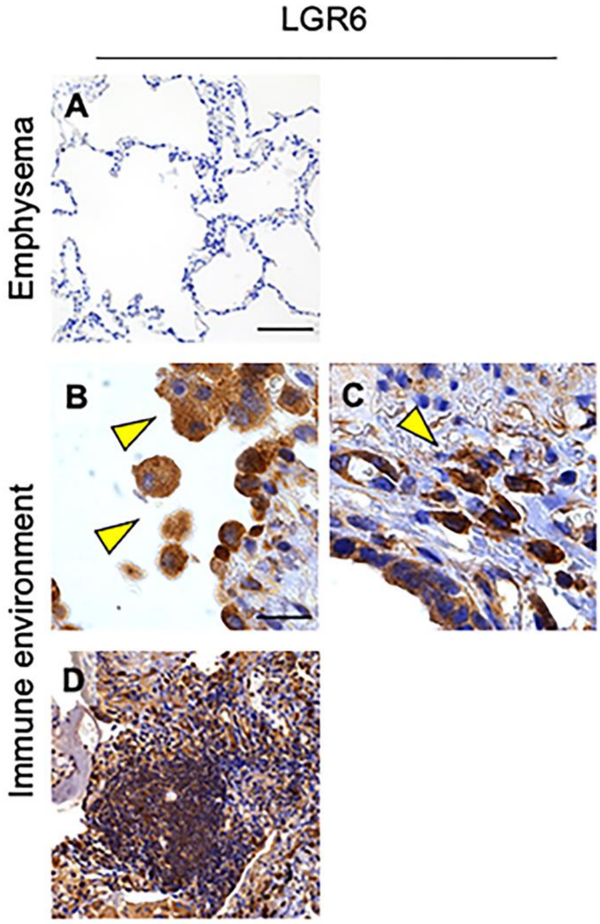

Figure S1. Expression of LGR6 in the tissue environment. **A:** In COPD samples, emphysema lesions were extensively observed in alveolar regions. Emphysematous areas were negative for LGR6 expression (A; bar = 100µm). **B-D**: In contrast, both in IPF and COPD, medium to high levels of LGR6 were reported in alveolar (B) and interstitial (C) macrophages, respectively (arrows; bar = 20µm). Moreover, increased LGR6 expression was observed in cells of lymphoid follicles of COPD and IPF lungs (D; refer to bar in Supplementary Figure 1A).

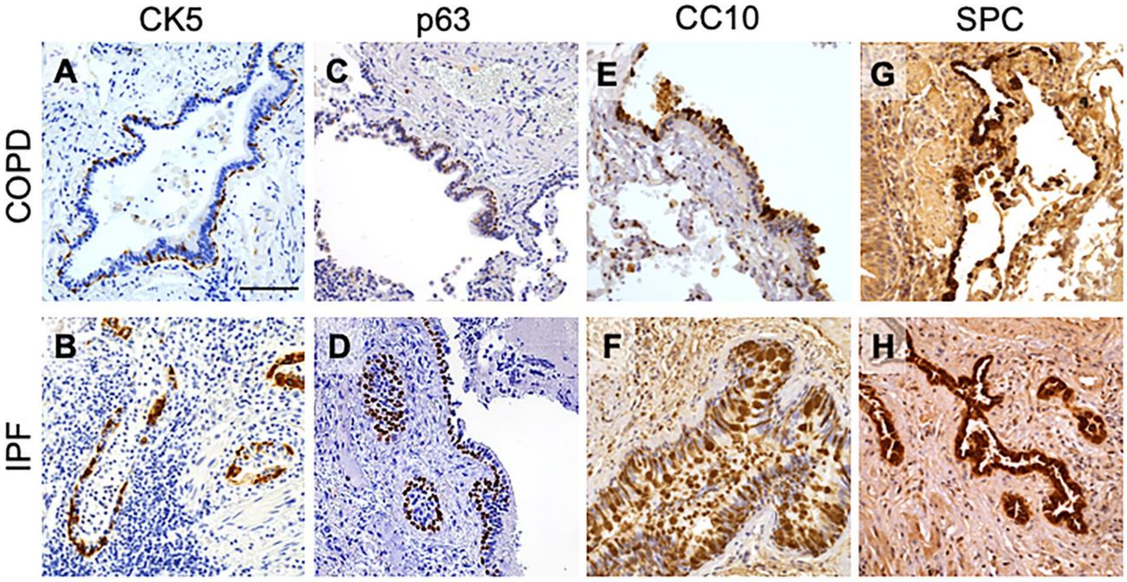

Figure S2. Alteration of progenitor cells niches in the lungs with COPD and IPF. **A-H**: Bronchiolar and alveolar compartments present profound rearrangements in COPD and IPF. Heavily damaged bronchioles showed loss and/or altered localization of basal progenitors (A-D; bar = 100µm), club cells (E-F) and ATII cells (G-H).

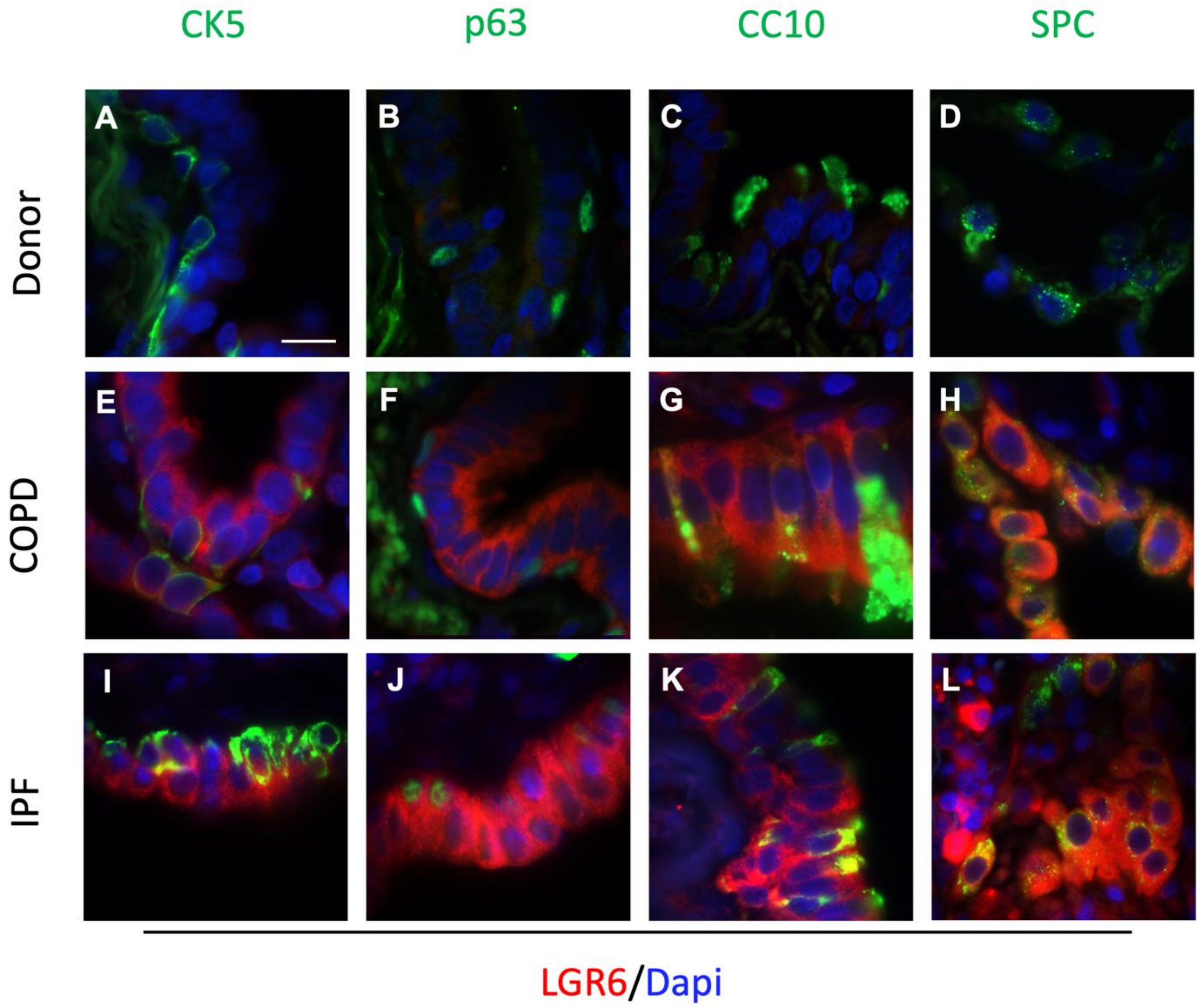

Figure S3. LGR6 expression in progenitor populations. **A-L**: Immunofluorescent stainings of Donor, COPD (A-H) and IPF (I-P) tissues revealed co-expression between LGR6 and epithelial progenitor markers CK5, p63, CC10 and SPC (bar = 20μm).

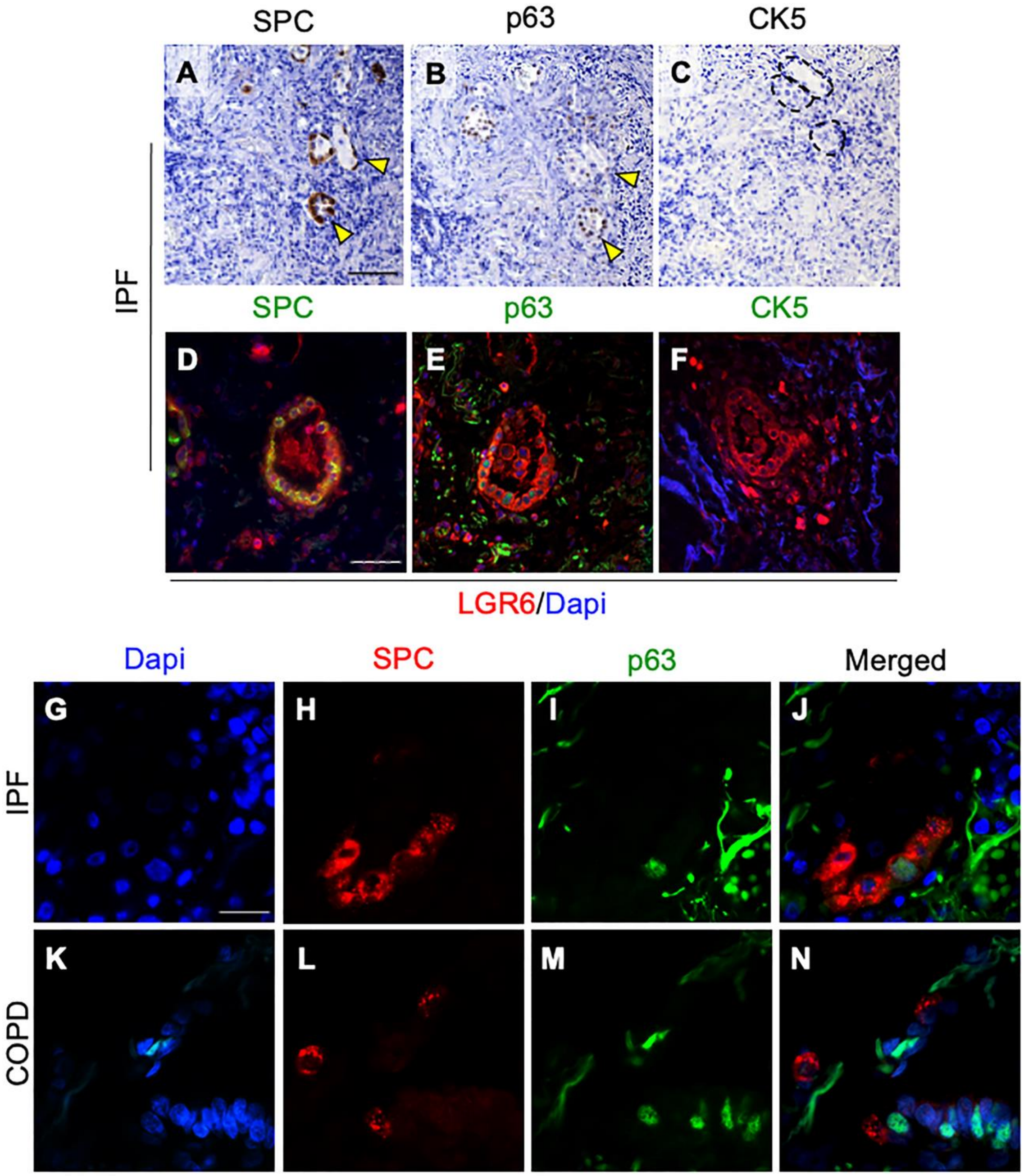

Figure S4. LGR6 in alveolar and basal progenitors. **A-F**: In consecutive slides, at the bronchioles-alveoli interphase, narrowed alveolar regions showed expression of SPC and p63, but not CK5 (A-C; arrows and lines, bar = 100μm). Co-expression between $LGR6^+/SPC^+$ and $LGR6^+/p63^+/CK5^-$ cells was observed in these lesions (D-F; bar = 50μm). **G-N**: SPC and p63 were co-expressed in few cells of narrowed alveolar IPF lesions (G-J; bar = 20μm). Only in rare occasions, co-expression between SPC and p63 markers was reported (K-N).

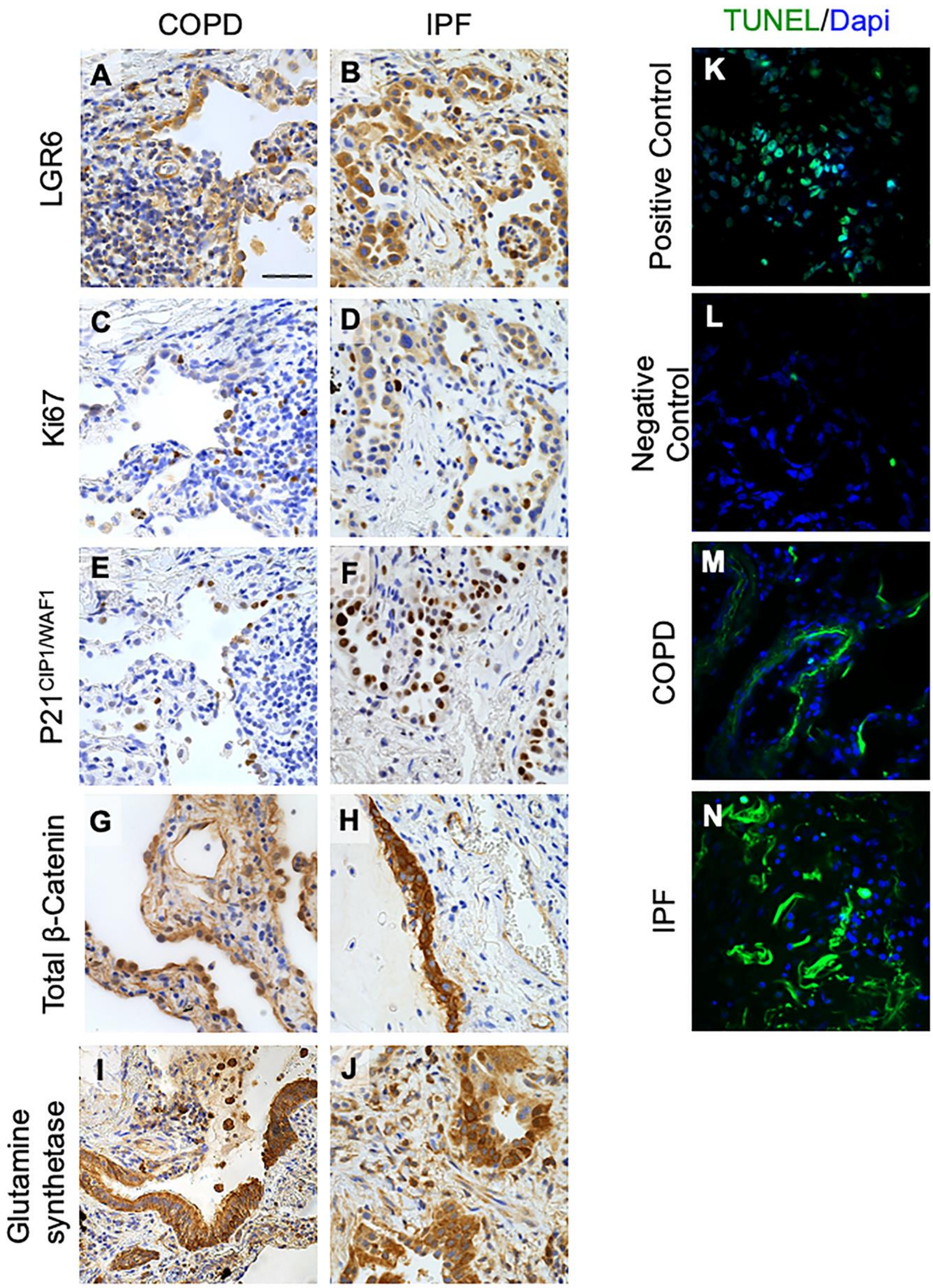

Figure S5. Non proliferating LGR6+ cells express markers of senescence and Wnt pathway activation, but not markers of apoptosis. **A-J**: In COPD and IPF, high levels of LGR6 (A,B; bar = 50μm) are associated with low proliferation (C,D), and increase in expression of senescence-associated (E,F) and Wnt-related (G-J) markers in epithelial progenitors. **K-N**: In situ apoptosis was evaluated through TUNEL assay, but no positive epithelial cells were detected (bar = 50μm).

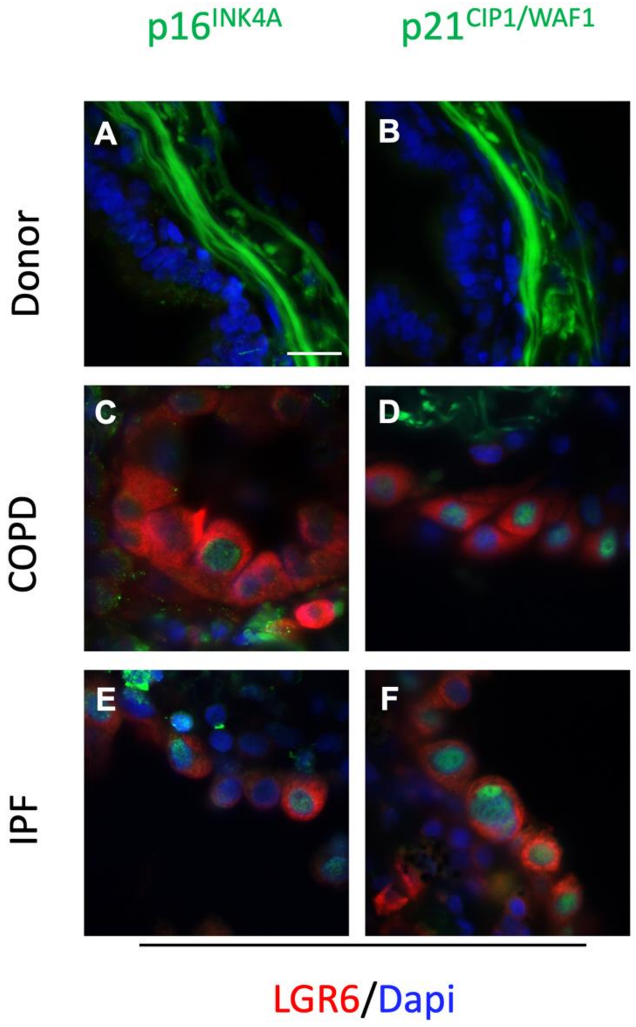

Figure S6. Senescent LGR6+ cells accumulate in COPD and IPF tissues. **A-F**: Immunofluorescent stainings of Donor, COPD (A-H) and IPF (I-P) tissues shows expression of $p16^{INK4A}$ and/or $p21^{CIP1/WAF1}$ in LGR6-positive cells (bar = 20μm).

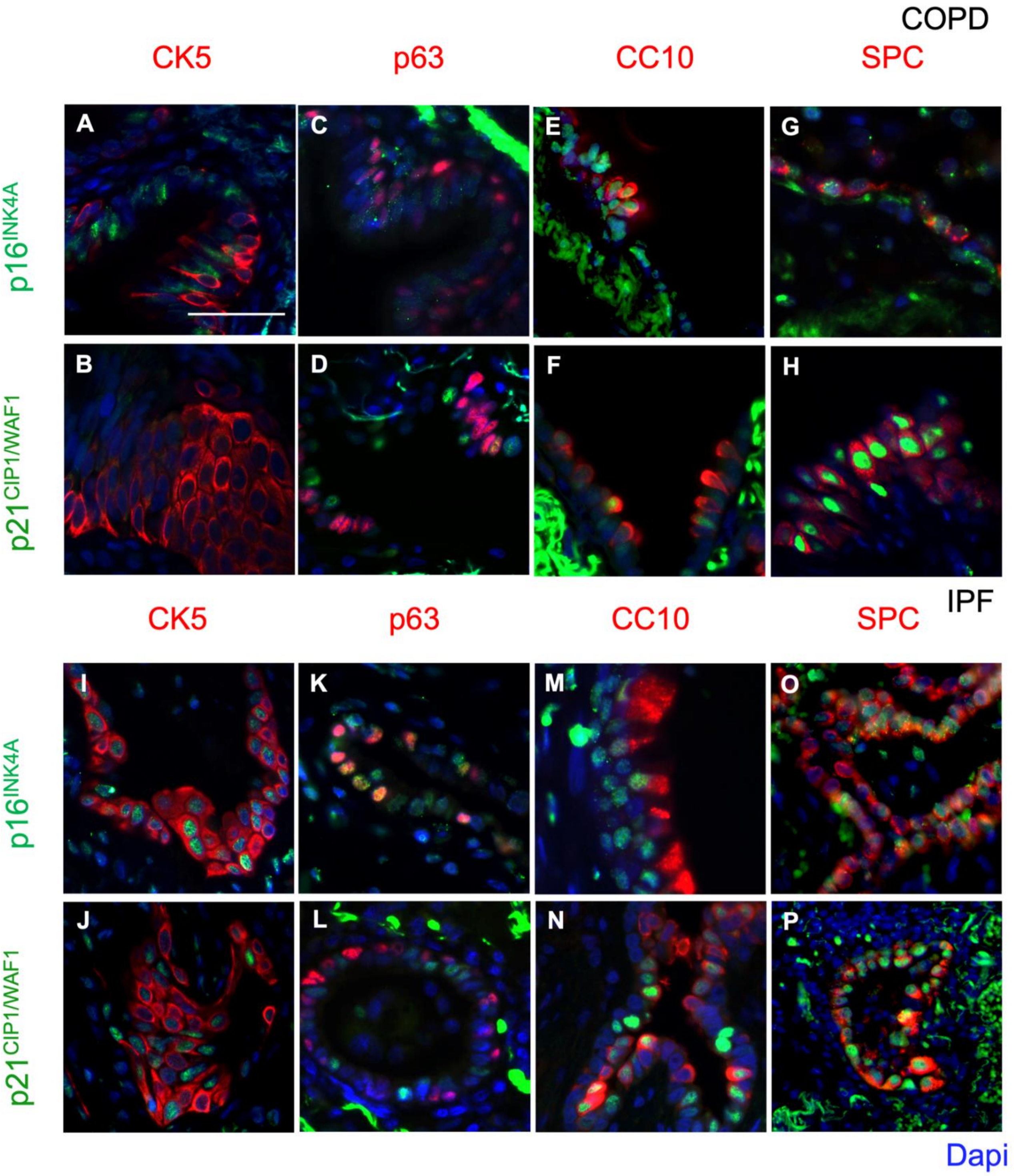

Figure S7. Lung epithelial progenitors show expression of senescence-associated markers. **A-P**: Immunofluorescent stainings of COPD (A-H) and IPF (I-P) tissues revealed co-expression between epithelial progenitor markers and $p16^{INK4A}$ and/or $p21^{CIP1/WAF1}$ (bar = 50μm).
